# Determination of Radical Scavenging Activity and Total Phenols of Wine and Spices: A Randomized Study

**DOI:** 10.3390/antiox2030110

**Published:** 2013-07-25

**Authors:** Fulgentius Nelson Lugemwa, Amanda L. Snyder, Koonj Shaikh

**Affiliations:** Department of Chemistry, Pennsylvania State University-York, 1031 Edgecomb Avenue, York, PA 17403, USA; E-Mails: als5216@yahoo.com (A.L.S.); kys5238@psu.edu (K.S.)

**Keywords:** antioxidant, spice extracts, wine, DPPH, total phenols

## Abstract

Thirty eight bottles of red wine (Carbanet Sauvignon) were randomly selected based on vintage, region, price, and age (number of months in a barrel). The total phenolic content of each wine was determined using Folin-Ciocalteau assay. The radical scavenging activity was evaluated using 2,2-diphenyl-1-picryhydrazyl (DPPH) assay. Apart from a few bottles that exhibited above average radical scavenging activity and phenolic content, there was no good correlation of those two quantities with region, price or vintage. The average phenolic amount was 2874 mg/L. The lowest phenolic content was found to be 1648 mg/L for an eight dollar wine. Wine with the highest amount of phenol of 4495 mg/L was a 2007, nine dollar bottle from South America. High amount of phenols did not translate into high radical scavenging activity. Barrel-aging did not increase the amount of phenols or the radical scavenging activity of wine. In order to discover new and potent sources of antioxidants from plants, the following spices were studied: ginger, cilantro, cumin, anise, linden, eucalyptus, marjoram, oregano, sage, thyme and rosemary. Whole spices were crushed and extracted for 96 h at room temperature using a combination of ethyl acetate, ethyl alcohol and water in the ratio of 4.5:4.5:1 (v/v/v). The radical scavenging activity of extracts was evaluated using 2,2-diphenyl-1-picryhydrazyl (DPPH) assay. The total phenolic content of each spice was also determined using the Folin-Ciocalteau assay. Eucalyptus was found to be the most potent antioxidant with an LC_50_ of 324.1 mg of phenol/L, followed by marjoram with an LC_50_ of 407.5 mg of phenol/L, and rosemary with an LC_50_ of 414.0 mg/L. The least potent antioxidants were ginger and cilantro with LC_50_ of 7604 mg/L of phenol and 7876 mg of phenol/L, respectively.

## 1. Introduction

Free radicals are naturally produced under aerobic conditions and are part of normal physiological processes, however, an excess of free radicals can damage all cellular macromolecules including proteins, carbohydrates, lipids, and nucleic acids [1]. The free radicals initiate reactions such as the oxidation of DNA, which can ultimately cause mutations in the genetic material and possibly cancer [2]. The oxidizing nature of free radical can result in enzymes inhibition or cause proteins to denature or degrade [3]. Epidemiological studies have consistently shown a link between the effects of free radicals and the onset of diseases such as cancer, arthritis, cardiovascular diseases, and advanced aging in humans [4,5]. Antioxidants are substances that neutralize the harmful free radicals in our bodies. Antioxidants act as “free radical scavengers” and hence prevent or slow the damage done by these free radicals. Antioxidants function as reducing agents, ultimately removing free radical intermediates and preventing further oxidation. Fruits and vegetables are known as good sources of antioxidants, such as retinol (Vitamin A), ascorbic acid (Vitamin C), α-tocopherol (Vitamin E), carotenoids, flavonoids, tannins, and other phenolic compounds [6,7,8,9]. Wine also contains antioxidants that have a protective and preventative effect caused by free-radical oxidations. It has been demonstrated that eating fresh fruits and vegetables and drinking two glasses of wine per day are some of the importance factors that contribute to living a healthy life. The lower incidence of cardiovascular disease mortality in southern France than in other European countries, despite a high consumption of saturated fats, was correlated with an increased intake of red wine [10,11,12]. Wine contains many polyphenolic compounds that include (+)-catechin, quercetin, and resveratrol, which are powerful antioxidants that can prevent *in vitro* and *in vivo* low-density lipoprotein free radical-mediated oxidation [13,14,15,16].

Antioxidants are also used to preserve food quality mainly because they slow oxidative deterioration of lipids. Much research is focused on finding ways to prevent or to delay the oxidation of fats and oils, which leads to deterioration of foods containing those components. Even small amounts of oxidizing free radicals can cause reactions that produce undesirable flavor, rancid odor, discoloration and other spoilage. Lipids that contain large amounts of unsaturated fatty acids can be easily oxidized to lipid peroxide or hydroperoxide, which then rapidly decomposes by radical chain reaction to give lower molecular weight compounds such as aldehydes, ketones, carboxylic acid and others. These compounds contribute to food rancidity. Various efforts have been made to minimize the oxidation reactions in foods such as reducing the amount of oxygen in the head space of containers, filling with inert gas, or addition of antioxidants. The addition of antioxidants to food is an effective way to prevent the development of various off-flavors and undesirable compounds that result from lipid oxidation. There has been a decreased use of the stable and effective synthetic antioxidants such as butylated hydoxytoluene (BHT) and butylated hydroxyanisole (BHA) because of the possibility of toxicity as well as rejection by the general consumer. As a result, there is worldwide interest in finding new and safe antioxidants from natural sources. The development of safe and effective antioxidants is focused on edible plants, especially spices, because man has used them not only as flavoring agents but also for antiseptic or medicinal purposes since prehistoric era. Natural antioxidants have the potential to prevent not only lipid peroxidation in food but also oxidative damage to living cells. Many spices have been examined for their antioxidative activity. Rosemary, sage, oregano, thyme, and turmeric were found to be effective antioxidants and some retarded the oxidation of lard [17,18]. The practical effectiveness of ground spices was examined in different types of food [19]. Other studies focused on the antioxidative effect of spice extracts [20,21,22,23]. Those results led to attempts to extract pure, active components. Rosemary is one of the most effective spices used in food processing and several active compounds have been isolated from its leaves [24,25,26,27]. Antioxidants have also been isolated from oregano [28], sage [29], and ginger [30].

Both wine and spices are consumed in large amounts daily worldwide and are of interest as potent sources of antioxidants. A bottle of wine, bought from a liquor store, is usually consumed on the same day, and the number of people drinking wine is on the rise because wine is thought to have health benefits. In this report, our interest was to discover the type of Carbanet Sauvignon wine with the highest free radical scavenging activity. Cabernet Sauvignon is one of the world's most widely recognized red wine grape varieties. It is also grown in nearly every major wine producing country among a diverse spectrum of climates. In addition, in an attempt to come up with safe and effective antioxidants that could be used in the food industry, to delay and prolong the shelf-life of various products that contain lipid, spices that not only flavor food but also are consumed for their medicinal purposes were evaluated.

## 2. Experimental Section

### 2.1. Reagents and Materials

Cabernet Sauvignon from Argentina, Australia, California, Chile, France, and South Africa was purchased from liquor stores in central Pennsylvania. McCormick, Clover Valley, Badia, and Price Rite spices were purchased from grocery stores in the same area. Spice Latin names were not listed on spice containers. Optical densities were read using Cole Parmer Instrument (Cole Palmer Company, Vernon Hills, IL, USA) 2800 UV/Vis spectrometer. 2,2-Diphenyl-1-picrylhydrazyl (DPPH), sodium carbonate, potassium acetate were purchased from Sigma-Aldrich, St Louis, MO, USA.

### 2.2. Determination of Total Phenolics in Wines

Determination of total phenolics as gallic acid equivalent was carried out using Folin-Ciocalteau method with minor modifications [31]. Wine (2 μL), distilled water (1.58 mL), Folin-Ciocalteau reagent (100 μL), and sodium carbonate (300 μL) were mixed and incubated at 40 °C. After 30 min, optical density was read at 765 nm, and the amount of total phenol calculated using gallic acid calibration curve.

### 2.3. Measurement of the Free Radical-Scavenging Activity in Wine

The free radical scavenging activity was measured by the 2,2-diphenyl-1-picrylhydrazyl (DPPH) method described by Blois, with some modification [32]. Stock solutions of wine were made by diluting 5 mL of wine with 10 mL of 13.5% neutral alcohol. Diluted samples of 5, 10, 15, 20 and 25 μL were mixed with DPPH (2900 μL of 0.03 mg/mL solution). Sufficient methanol was added to each sample to obtain a total volume of 3 mL. After standing for 30 min at room temperature, the absorbance was measured at 517 nm. High absorbance of the reaction mixture indicated low free radical scavenging activity. The volume of wine in the diluted solutions needed to decrease the initial DPPH concentration by 50% together with the amount of phenol in mg/L were used to obtain the LC_50_ values in mg of phenol/L. The capability to scavenge the DPPH radical was calculated using the following equation:

*I* = [((OD_o_ − OD_s_)/OD_o_) × 100] (1)

where *I* was the inhibition percentage; OD_o_, was the absorbance of the negative control (containing 100 μL of MeOH instead of the sample); and OD_s_ was the absorbance of the samples. The percent inhibition was plotted against volumes of wine using Microsoft Excel and the volume needed to decrease DPPH concentration by 50% was calculated from the graph (*R*^2^ ≥ 0.95). The experiment was carried out in triplicate and the results are mean values.

### 2.4. Extraction of Spices

Spices were dried at 75 °C in oven to constant weight. Whole dried spice (1 g) was crushed and extracted for 96 h at room temperature using a combination of ethyl acetate, ethyl alcohol and water (20 mL) in the ratio of 4.5:4.5:1 (v/v/v). This extraction process, developed in our laboratory [33], was exhaustive as subsequent extracts produced less 5% activity of the first extract as measured by the DPPH assay. Data not shown. Drying whole spices at 75 °C did not decrease the amount of phenolics or affect radical scavenging activity of spices.

### 2.5. Determination of Total Phenol in Spice Extracts

To each spice extract (20 μL) was added 1.58 mL water and 0.1 mL Folin-Ciocalteau reagent. After nine minutes, 0.3 mL of saturated solution of sodium carbonate was added and the mixture kept in a water bath at 40 °C. After 30 min, optical density was read at 765 nm and the amount of phenol calculated using amount of gallic acid per liter *versus* optical density calibration curve.

### 2.6. Measurement of Spice Free Radical-Scavenging Activity

Extracts of rosemary, eucalyptus leaves, and marjoram were diluted five times before carrying out the DPPH assay. In order to find the LC_50_, aliquots of original or diluted extract ranging from 10 μL to 80 μL were mixed with enough methanol and DPPH (2900 mL of 0.03 mg/mL solution) to give a total volume of 3 mL. The volume of extract needed to decrease the initial DPPH concentration by 50%, together with amount of phenol as measured by the Folin-Ciocalteau method were used to determine LC_50_ values.

## 3. Results and Discussion

### 3.1. Total Phenolic Compounds and Radical Scavenging Activity of Wines

Cabernet Sauvignon from the Australia, Chile, Argentina, Pennsylvania, California, South Africa, and France were used in two assays to determine the radical scavenging activity and total phenols. Table 1 shows the total phenol (mg/L) and the radical scavenging activity (RSA) in mg of phenol/L of wine from different regions, costing different prices, and having aged in a bottle for at least four years at the time of this study. Alcohol (13.5%) was used to dilute wine in order to maintain the original alcohol content in the wine during the assay. Total phenols as determined by the Folin-Ciocalteu method varied from 1648 mg/L to 4495 mg/L, GAE, with the average of 2874 mg of phenol/L. Radical scavenging activity as measured by the DPPH assay varied from 1.00 mg phenol/L to 1.90 mg of phenol/L with the average LC_50_ of 1.60 mg of phenol/L. Wine from California, entry 1–10, generally exhibited good total phenol numbers ranging from 1648 mg/L to 3475 mg/L. Their overall RSA was slightly higher than the average for all wines. An eight dollar bottle, entry 5, with the lowest amount of phenols of 1648 mg of phenol/L had the highest RSA with an LC_50_ of 1.0 mg phenol/L. Wine from Argentina had consistently higher levels of phenols and lower RSA than wine from California. One bottle of wine from Argentina had the highest amount of phenols, entry 23. However, the radical scavenging activity was found to be one of the lowest with an LC_50_ of 1.9 mg phenol/L. Each region had wine with high phenol numbers and high RSA activity. At the same time, wine with low phenol numbers and low RSA relative to the phenols was also found in every region. Overall, high amounts of phenol did not produce high radical scavenging activity. This is not surprising because Folin-Ciocalteau reagent reacts with vitamins, thiols, dihydroxyacetone, inorganic salts, and nitrogen-containing compounds [34]. Some of those compounds are found in grape skin. Complexation with copper increases the reactivity of Folin-Ciocalteau reagent [35]. Moreover, the ability of phenolic compound to scavenge free radical depends on the structure of the phenol. Argentina wine had above average total phenols, however, that was not true with the radical scavenging activity. Some studies have found that the antioxidant activity of wines correlated well with the phenolic content [36,37], but one author reported no correlation between the total concentration of phenolic compounds and antioxidant activity of conventional and ecological red and white wines [38].

**Table 1:** Total phenolics and antioxidant activity of unaged wine.

Entry	Region	Vintage	Price $	Total phenols (mg/L)	LC_50_ (mg of phenol/L)
1	California	2005	40	2940	1.5 ± 0.2
2	California	2006	27	3475	1.4 ± 0.2
3	California	2007	9	2403	1.5 ± 0.2
4	California	2007	12	2658	1.6 ± 0.3
5	California	2007	8	1648	1.0 ± 0.1
6	California	2007	11	3094	1.6 ± 0.3
7	California	2007	14	2735	1.6 ± 0.3
8	California	2008	12	2312	1.5 ± 0.2
9	California	2008	6	2285	1.7 ± 0.2
10	California	2008	12	2467	1.6 ± 0.2
11	Chile	2007	10	2841	1.7 ± 0.3
12	Chile	2007	12	3910	1.9 ± 0.3
13	Chile	2007	11	2878	1.8 ± 0.3
14	Chile	2007	21	2874	1.7 ± 0.3
15	Chile	2008	18	2678	1.7 ± 0.2
16	Chile	2008	10	2783	1.9 ± 0.3
17	Chile	2008	15	3001	1.8 ± 0.3
18	Chile	2008	10	2568	1.7 ± 0.2
19	Chile	2008	20	2456	1.8 ± 0.3
20	Chile	2008	15	2874	1.9 ± 0.3
21	Argentina	2007	20	2885	1.3 ± 0.1
22	Argentina	2007	11	3375	1.7 ± 0.2
23	Argentina	2007	9	4495	1.9 ± 0.2
24	Argentina	2008	11	3299	1.6 ± 0.2
25	Argentina	2008	9	3456	1.7 ± 0.2
26	Australia	2007	10	3621	1.9 ± 0.3
27	Australia	2007	15	2185	1.4 ± 0.1
28	Australia	2007	8	2739	1.7 ± 0.2
29	Australia	2007	15	2988	1.5 ± 0.1
30	Australia	2008	8	2345	1.7 ± 0.2
31	France	2006	8	3412	1.7 ± 0.2
32	France	2006	12	2849	1.4 ± 0.2
33	France	2007	16	3211	1.7 ± 0.2
34	France	2007	13	2789	1.7 ± 0.2
35	France	2007	12	2887	1.6 ± 0.2
36	South Africa	2006	10	2671	1.7 ± 0.3
37	South Africa	2007	10	2703	1.6 ± 0.2
38	South Africa	2007	10	2434	1.6 ± 0.2

The effect of barrel-aging on the total phenols and radical scavenging activity is shown in Table 2. There is no noticeable increase or decrease in the amount of phenols as a result of barrel-aging. Had there been appreciable extraction of tannins from wood, the total phenolics and RSA would have been higher. In this randomized study, it was not possible to compare one aged and one unaged sample of the exact same wine. Having one sample put in a barrel and another one kept in a bottle would have provided a better way of evaluating the effect of barrel-aging. However, by comparing data in Table 1 with that in Table 2, the evolution of polyphenols in samples that were not barrel-aged seem to proceed at the same rate as in barrel-aged samples. The phenolic content of wine kept in bottles, Table 1, and those kept in barrels, Table 2, are in the same range. Unlike in barrel-aging of whiskey, where the alcohol content is high and the aging period longer, resulting in significant extraction of tannins, the tannin extraction in wine seemed negligible. The radical scavenging activity of the whiskey also increased with the aging period with high correlation [39,40]. The wine alcohol content is low and the time the wine spends in barrels is short. One of the reasons for keeping wine in barrels is to achieve smoothness and roundedness, and to get some additional flavors from wood. The amount of “harsh” tannins that are removed by adsorption or any tannins extracted from the barrel must be small compared to the total phenolic content the wine gets from the grapes. Moreover, enological tannins are routinely added to wines, and if the amount added is much greater than the amount extracted from wood, any slight increase due to extraction can be difficult to measure. The radical scavenging activity of wine kept in barrels did not seem to depend on aging time. Again, without identical samples, some put in barrels and others put in bottles and monitored at regular intervals, it was impossible to evaluate the effect of barrel-aging with time. It would have been helpful to have established a starting baseline, but that was not possible in this randomized study. However, by looking at the RSA activity of barrel-aged wine, Table 2 and that of bottle-aged wine, Table 1, there was no dramatic difference observed.

**Table 2:** Total phenolics and radical scavenging activity of barrel-aged wine.

Region	Vintage	Months in barrels	Total phenols mg/L	LC_50_ mg of phenol/L
California	2005	24	2987	1.9 ± 0.2
California	2005	24	3005	1.7 ± 0.2
California	2006	14	3578	1.5 ± 0.1
California	2007	14	2398	1.3 ± 0.1
California	2007	14	2398	1.5 ± 0.1
Pennsylvania	2011	11	3132	1.7 ± 0.2
Pennsylvania	2011	11	2879	1.6 ± 0.2
Pennsylvania	2011	11	2856	1.8 ± 0.2
Chile	2007	10	4011	1.9 ± 0.2
Chile	2007	10	3450	1.7 ± 0.2
Argentina	2007	4	2995	1.5 ± 0.2
Argentina	2007	4	2902	1.3 ± 0.1

### 3.2. Total Phenolics and Radical Scavenging Activity of Spice Extracts

Each spice was obtained from four different suppliers, namely, McCormick, Price Rite, Badia, and Clover Valley. Leaves of spices were used for all plants except for cumin and ginger; seeds and roots were used for cumin and ginger, respectively. The total phenols and LC_50_ for each spice given in the table are averages from twelve different determinations. Total phenolic content was expressed as mg gallic acid equivalents (GAE)/g. Linear regressions were performed to obtain LC_50_ values using Word Excel. The data in Table 3 show that the phenolic content varied from 191.8 mg/L in anise to 4033 mg/L in oregano. The radical scavenging activity varied from 324.5 mg phenol/L found in eucalyptus to 7880.2 mg of phenol/L in cilantro. Eucalyptus was the most potent antioxidant. Marjoram and rosemary had more or less similar radical scavenging activity. Thyme, oregano and sage had moderate activity; and linden leaves, anise, cumin, ginger, and cilantro had the lowest activity. In general, correlation between the amount of phenolic compounds as measured by GAE and antioxidant activity was observed. Spices with high amount of phenol exhibited good antioxidant activity. A number of authors have isolated and examined antioxidants from rosemary [41,42,43,44,45,46], oregano [28,47] and sage [47,48,49]. Antioxidants in rosemary are attributed mainly to carnosic acid, carnosol and rosmarinic acid [50], but other phenolic compounds are also present in good amount [51]. Compounds from spices are quite complex and as such it is a challenge to isolate all active compounds from a given spice. We reasoned that using a total extract in the assay was a good way to assess the antioxidative properties of all extractable compounds. Isolating pure compounds is not only tedious and might lead to loss of active components, but it also eliminates any synergism that might exist among different active components. Also using total extracts as food or preservative could prove to be less costly.

**Table 3:** Total phenolics and radical scavenging activity of spices.

Spice	Total phenol (mg/L)	LC_50_ (mg of phenol/L)
Eucalyptus	1387.5 ± 10.5	324.5 ± 15.5
Marjoram	1159.0 ± 9.2	408.0 ± 10.3
Rosemary	1972.3 ± 7.5	414.2 ± 14.6
Thyme	1987.3 ± 5.2	484.3 ± 18.5
Oregano	4033.1 ± 10.1	592.5 ± 15.5
Sage	1212.5 ± 8.5	788.2 ± 10.2
Linden leaves	375.6 ± 7.8	3760.4 ± 101.5
Anise	191.8 ± 9.3	5140.7 ± 105.0
Cumin	220.0 ± 10.9	5910.9 ± 110.4
Ginger	556.5 ± 70.5	7600.5 ± 230.5
Cilantro	251.0 ± 32.3	7880.2 ± 118.2

## 4. Conclusions

What distinguishes a great wine from a good wine is usually balance, which takes into account aroma, bouquet, fruit flavors, body, structure and mouth-feel. In this study, cheap wine was found to have more or less the same radical scavenging activity as the more pricey wine. The amount of total phenols as measured by GAE was also comparable. Our study indicates that there isn’t a major difference between wine from different regions based on total phenolic content and radical scavenging activity. Barrel aging that softens tannins and contributes to the overall roundedness did not seem to affect the total phenols or the radical scavenging activity of wine. Without blind tasting, coupled with the measurement of total phenolics, it was not possible to correlate taste to amount of phenols. It is possible that wine producers make the right adjustments to wine before selling it to the public. Adjustments may include blending and addition of enological tannins from different sources. Future studies will take into account blind wine tasting and the measurement of phenols and radical scavenging activity to evaluate the gradual evolution of phenolics.

Rosemary, eucalyptus, marjoram, and thyme, all had good radical scavenging activity compared to other spices. Rosemary has a unique aroma and has been used in food preservation. Our study indicates that eucalyptus leaves, marjoram and thyme could be good substitutes for rosemary, expanding the aroma choice, as each spice has its own characteristic smell. The total phenolics in wine and in the spices we examined in this study are in the same range, however, the radical scavenging activity of wine is about two hundred times that of eucalyptus, the spice with the highest radical scavenging activity. Taking two glasses of wine would provide more antioxidants that eating a whole rosemary-flavored chicken.
